# Syphilis as Seen by the Dentist

**Published:** 1901-01-15

**Authors:** Norton D. Coons


### SYPHILIS AS SEEN BY THE DENTIST.

BY NORTON D. COONS, M.D., D.D.S.

Read before the Washtenaw County, Michigan, Dental Society.

In preparing this paper I have endeavored to confine myself as much as possible to those phases most likely to be seen by the dentist.

Syphilis is many times an important cause of those diseases which baffle the skill of the dentist to satisfactorily diagnose or cure, and by taking it into account no doubt prolonged treatments with unsatisfactory results would often be avoided.

It is an infectious disease propagated either by inoculation or by hereditary transmission. Its specific microorganism has not so far been isolated. In 1884 and 1885 Lusgarten published a method for staining bacilli found in syphilitic tissue, and which he assumed to be the cause of the disease. Other observers have since found the bacilli, but as yet there is no proof that it is the specific organism.

Syphilis is a disease peculiar to man, the lower animals are not affected by it. It is usually acquired through sexual intercourse; it may be acquired by accidental inoculation. Hence the practical necessity for a dentist being able to recognize its manifestations in the mouth, that he may take extra precaution in the sterilization of any instrument used on such patients.

Should an infected excavator wound the mucous membrane of an innocent patient, a primary lesion might occur.

Hereditary transmission is often observed, and is of much interest to the dentist from its liability to interrupt the normal calcification of the permanent teeth.

In the acquired form, in about three weeks from the time of infection the primary lesion or chancre develops at the point of inoculation, and nowhere else. It usually begins as a small papule, which gradually enlarges, or by a superficial fissure. In either case there is an induration of the tissues at the point of attack. As it enlarges the epithelium desquamates, leaving a red surface upon the mucous membrane like a superficial abrasion or ulceration covered by a grayish or yellowish false membrane.

The induration observed is more or less marked, sometimes giving the sensation of a hard nodule, fibrous or cartilaginous in character, again resembling a thin plate of parchment paper. It is caused by a sclerosis or thickening of the walls of the arterioles and venules of the skin. This induration is usually seen about a week after the appearance of the chancre, and may remain at its height for three or four weeks, whilst the chancre may not heal for five or six weeks. If the nodule be large and cartilaginous it may be easily detected for many years.

Ulceration of a chancre when found is simply a cup-shaped depression, its surface smooth and its margins not abrupt. In its center is a false membrane, which, when removed, leaves a raw, bleeding surface. The general health is usually good at this stage, and a valuable symptom may be the painless enlargement of the nearest lymphatic gland.

While the chancre is usually found on or near the genitals, it often appears on the lips, cheek, tonsils, tongue or palate, and may be mistaken for an epithelioma.

The following comparative statement will enable a correct differential diagnosis to be made:

| LABIAL CHANCRE. | EPITHELIOMA. |
|---|---|
| No marked difference in sexes. | Much more common in males. |
| May involve either lip. | Usually located on lower lip. |

| LABIAL CHANCRE. | EPITHELIOMA. |
| --- | --- |
| Occurs at any age. | Rarely before middle life. |
| Patient strong and robust. | Usually health impaired. |
| Is not sensitive. | Often sharp, burning pains. |
| Regular outline, smooth, elevated surface. | Irregular, ragged outline and filled with fungus granulations which bleed easily. |
| Indurated and sharply circumscribed base. | Induration unequal but clearly circumscribed, but more extensive. |
| Evolution of sore usually occupies at least five weeks. | May be months in healing after first appearance. |
| Glandular enlargement soon after first appearance. | Glands not implicated for three or four months, or even later. |
| No marked odor from secretions of sore. | Odor often quite offensive. |
| History of exposure. | |
| Heals under mercurial treatment. | No effect, if any, only to make the difficulty worse. |

Microscopic examination of a piece of affected tissue will insure a correct diagnosis.

The small ulcers on the side of the tongue, produced by contact with rough or carious teeth, have been mistaken for chancres.

During the first few days the diagnosis may be difficult, but if the original papule or fissure enlarges, is indurated and elevated, the epithelial covering drops off, and the lesion becomes covered with a grayish, diphtheritic membrane, one should at least suspect syphilis. This may be confirmed later by the painless enlargement of the submaxillary glands. Silver nitrate will usually cure a simple ulcer on the tongue, but has no effect on the chancre.

Six weeks to three months after the first appearance of the chancre other symptoms known as secondary symptoms begin to appear. There has been a gradual enlargement of the lymphatic glands, and now all the glands of the body show this enlargement. Together with glandular enlargement certain constitutional symptoms are observed. First. An irregular fever, noticed especially at night, and varying from 100 to 101 degrees. Second. Muscular and arterial pains affecting chiefly the muscles of the chest and back and the joints of the arms. Third. Alopecia, affecting the hair of the entire body, distinguished from ordinary baldness by a similarity to appearance of a moth-eaten garment, and other syphilitic appearances.

The secondary eruptions appear at this time and may be seen on the skin and mucous membranes. The earliest cutaneous symptom makes its appearance as an erythematous or rose color rash. The former appears as a diffuse mottling of the skin, affecting chiefly the trunk and abdomen, and is without appreciable elevation. The latter is darker in color, and has a tendency to become papular. The older lesions are larger and deeper.

A syphilitic papule located on the skin has a dry, desquamating surface, but if situated on a moist surface, as in a fold of skin or on the mucous membrane, it is converted into what is known as a *mucous patch*. In such case the epidermis is macerated and on a mucous membrane it has a grayish appearance, resembling mucous membrane touched with nitrate of silver. If it is on the skin it is red, smooth and polished. But in either case it is moist, has a secretion, which is usually offensive, and is always highly contagious.

The color of a syphilitic papule is characteristic, and is said to resemble copper in color, owing to the extravasation of red blood corpuscles into the tissues, and appears in the earliest eruptions. A papule may increase until it reaches a diameter of an inch. They retain in all cases a round shape and the copper color. Pustules also form, which leave scars on healing.

The period of secondary syphilis may last from one to three years, which may be followed by one which is known as the *intermediate period*, during which very slight symptoms are manifested or perhaps none at all. The time of this period varies lasting from two to four years, or end in recovery, or pass to the *tertiary period*, lasting indefinitely.

During the *intermediate period* the patient is immune to fresh infection, but children begotten during this time are likely to suffer.

The lesions of the third stage are circumscribed tumors known as *gummata*. They are hard and firm—a small gumma, histologically, is made up of granulation tissue. Owing to meager blood supply coagulation necrosis takes place in the center of the gumma, and a cross section of a medium sized gumma shows a caseous material in the center. They may be found in the bones, periosteum, muscles, skin, brain, etc. Those of the mouth are of most interest to the dentist. They are found on the mucous membrane of the tongue, soft palate and pharynx. They are usually ulcerative and localized. They do not spread much, but involve the deeper structures.

Gummata of the tongue may be either submucous or muscular. The submucous gummata may be the size of a pea, and either single or multiple. They begin as small, hard nodules, softening later, discharging through a small aperature.

Muscular gummata are larger reaching the size of a hazel nut, and occupy the lateral or median aspects of the tongue, but may affect either the tip or base. At first these open by a narrow channel which enlarges and extends along the muscle fibers.

In these affections of the tongue it is rare to find enlargement of the cervical or submaxillary lymph glands; the same is also true of gummata of the pharynx. These gumma are sometimes mistaken for epithelioma or tuberculous ulcers. From the character of the ulcers alone it would be almost impracticable to differentiate them. The tubercular ulcers often show a fine yellow point with an opaque center, which are tuberculous granulations undergoing caseous degeneration, eventually to be thrown off by the ulcerative process. These are not seen at all in syphilis. The gumma begins as a single mass, opening after a time by a contracted passage, with an ulcerative discharge and a sloughing base.

The tubercle begins on the surface as a small nodule, and by the union of several a larger, irregular ulcer is formed. The ulcer has jagged edges and suppurates less than the gumma.

A chest examination might show pulmonary tuberculosis, or a syphilitic history would help to establish the probable gumma.

Epithelioma of the tongue can best be diagnosed by making a microscopic examination of a section of the suspected tumor. Nests of epithelial cells will appear.

The course of an epithelioma is much more rapid than that of a gumma. It many times runs its course and kills the patient in from twelve to eighteen months.

Gumma of the soft palate will perforate the bone unless the disease is stopped. These form slowly and grow insidiuously, and with little or no pain. The soft palate becomes red, thickened and nodular, at some point or entirely, and the induration can be felt with the finger.

A valuable diagnostic symptom is the immobility of the soft palate. This may be noted on requesting the patient to utter some sound requiring the elevation of the soft palate, as *oh*, when there will be an incomplete elevation or none at all. This symptom, the induration, prominence and thickening of the gumma often make an early diagnosis possible.

If left without treatment and both surfaces of the palate are affected, perforation may take place in so short a time as a single night.

Syphilis of the Bones.—The bones may become diseased during any stage of syphilis. The determining cause and seat in secondary and tertiary syphilis is usually some form of traumatism; it may be slight but frequently repeated at a single point.

Syphilitic bone lesions vary from simple periostitis and osteitis to gummata and the final consequences, from the development of exostosis to necrosis and formation of sequestræ in pus cavities.

The superficial bones being more exposed to injury are most often affected.

In syphilitic osteo-periostitis the pain and swelling are the chief symptoms. The pain being worse at night and intense at times, a very slight touch often producing great suffering. Potassium iodid administrated for two or three days will usually greatly relieve these symptoms, if not cure entirely.

The symptoms of syphilitic osteitis are variable and dependent often on the bones affected, as well as the form of the lesion. The nodular swelling, single or multiple nodules of varying size, and pain on pressure are usually present. The pain occurs spontaneously during the early part of the night.

*Treatment.*—As a rule, treatment should not be begun early in the first stage unless absolutely sure of the diagnosis. Should the case not be one of syphilis and syphilitic treatment be used, and the patient recover under such treatment, the result is likely to leave sufficient doubt about the case to affect the future life of the patient.

*Mercury* retards the appearance of secondary symptoms or prevents them altogether, so in doubtful cases it may be well to delay this treatment. Patients should be informed that treatment will be prolonged often for two or three years. Two years of mercury and at least six months of the mixed treatment (mercury and iodid of potassium).

The most satisfactory method of administering mercury is by the mouth, preferably the *protiodid of mercury*, in doses of from one-fourth to one-third of a grain in pill form three times a day, being careful not to give it in connection with iodid of potassium. If this should produce gastro-intestinal disturbances combine with it one-twentieth to one-twelfth of a grain of opium. Should this not overcome the action on the bowels, substitute *mercury with chalk* in one grain doses from four to six times each day. Half a grain of Dover's powder added to each dose will control any tendency to diarrhœa.

At the end of two years *potassium iodid* should be added and the mixed treatment continued for six months. The patient should then be kept under continuous watch for another year, without treatment, and should no symptoms reappear a cure may be pronounced. Should the symptoms recur, the patient should at once be put under treatment for another six months after the subsidence of all symptoms.

In obstinate tertiary syphilis potassium iodid may be given in from twenty to sixty grains three times each day. It should be well diluted to prevent its irritant action on the gastro-intestinal tract.

The local treatment is usually of minor importance. Mucous patches may be touched with silver nitrate or copper sulphate. A mouth wash of potassium chlorat to prevent salivation is also useful.

Hereditary Syphilis.—A child may acquire this disease either from the father or mother, but usually only during the early stages of the disease. Probably one-third of the syphilitic children are still-born, and one-fourth die within six months after birth.

Of course the primary stage is absent, but the secondary stage appears within from one week to three months after the child is born. When present at birth they consist largely of a withered or atrophied appearance of the child, accompanied by a hoarse cry, due to a swollen or even ulcerated condition of the laryngeal mucous membrane and a coryza due to an inflammation of the Schneiderian membrane. Cutaneous manifestations are also present.

The coryza is one of the most characteristic symptoms. Owing to an excess of blood supply a catarrhal condition is established, accompanied by a watery discharge, which dries and forms crusts which cause a peculiar nasal respiration known as the *snuffles*. Mucous patches are an early lesion and constitute one of the most frequent means of transmitting the disease to nurses and others with whom the child may come in contact.

Hereditary syphilis is of interest to the dentist because of its influence in deforming the permanent set of teeth. The teeth of the first dentition show only signs of an interference with nutrition, such as opaque and chalky enamel, soft, friable dentin and tendency to decay. The permanent dentition may likewise be affected, but this is associated with various cachexia and therefore not characteristic of syphilis.

The teeth that are characteristic and known as "Hutchinson's teeth" are often described as pegged, having the appearance of a row of pegs inserted into the gums. They are stunted, narrowing mesio-distally toward the incisal edge which is deeply notched. This refers more particularly to the upper incisors. All the teeth are separated and many varieties of abnormal forms are observed.

*Bibliography.*—The American Text-Book of Surgery, and Osler's Practice of Medicine.

